# Circulatory miR-223-3p Discriminates Between Parkinson’s and Alzheimer’s Patients

**DOI:** 10.1038/s41598-019-45687-x

**Published:** 2019-06-28

**Authors:** Roberta Mancuso, Simone Agostini, Ambra Hernis, Milena Zanzottera, Anna Bianchi, Mario Clerici

**Affiliations:** 1 IRCCS Fondazione Don Carlo Gnocchi, Milano, Italy; 2 Department of Pathophysiology and Transplantation, University of Milano, Milano, Italy

**Keywords:** Molecular neuroscience, Neurodegenerative diseases, Predictive markers

## Abstract

MiR-223-3p is involved in the regulation of a broad range of cellular processes and in many types of pathological processes as cancer, autoimmune and inflammatory diseases. MiR-223-3p has been indicated as negative regulator of NLRP3 protein, a key protein of inflammasome. The chronic inflammasome activation, an underlying feature of neurodegenerative disorders, is induced by misfolded protein aggregates, including amyloid-beta and alpha-synuclein, resulting in pro-inflammatory cytokines secretion and propagating of neuroinflammation. The aim of the study was to analyze whether circulatory miR-223-3p could be used as biomarker in neurodegeneration and to clarify its possible relationship with inflammasome activation. miR-223-3p concentration was evaluated in serum of Alzheimer’s (AD) and Parkinson’s disease (PD) or mild cognitive impairment (MCI) patients and healthy controls (HC). Compared to HC, miR-223-3p serum concentration was reduced in MCI and AD, but up-regulated in PD (p < 0.0001), and it decreased progressively from MCI to moderate (p < 0.0001) to severe AD (p = 0.0016). Receiver operating characteristic analysis showed that miR-223-3p concentration discriminates between AD, PD and MCI vs. HC, as well as between AD and PD. miR-223-3p serum concentration discriminates between AD/MCI and PD, suggesting that this molecule could be a potential non-invasive biomarker for differential diagnosis and prognosis of these neurodegenerative conditions.

## Introduction

The identification of circulating biomarkers allowing early diagnosis is a top priority to fight Alzheimer’s (AD) and Parkinson’s (PD) diseases, the most common age-associated neurodegenerative disorders in humans. In early stages the diagnosis of these diseases is specially challenging. Thus, even if evaluation of cerebrospinal fluid (CSF) levels of amyloid-β (Aβ), tau (T-tau), phospho-tau protein (P-tau) and α-synuclein (α-syn) can be a useful supports in clinical practice^1^, their use in the screening of patients is cumbersome due to the lumbar puncture invasiveness and the cost of these tests.

MicroRNAs (miRNAs) are small non-coding RNA molecules abundantly found within the nervous system, where they are key functional regulators of neuronal cells. Aberrant miRNAs expression has been observed in AD and PD brains, suggesting that different miRNAs which are known to regulate the expression of genes associated with neurodegeneration, can contribute to the pathogenesis of these diseases^2^. miRNAs regulate several biological processes inside the cell in which they are produced, but these molecules can also be packaged into extracellular vesicles so that they can be measured in extracellular fluids^3^. Notably, in this cell-free form, miRNAs can modulate target mRNA functions in other cells in an endocrine mode; several studies have identified the important role of miR-223-3p during intercellular communication, as miR-223-3p contained in microvesicles can attenuate inflammations and dampen injury in cells other than myeloid cells, like alveolar epithelial cells^4^.

Changes in the concentration of cell-free miRNAs can be detected in biological fluids of AD and PD patients^5^; these results support the idea that cell-free miRNAs could be used as biomarkers in these diseases. This possibility is extremely interesting given the fact that cell-free miRNAs can easily be quantified in serum/plasma with minimally-invasive procedures.

Different neurodegenerative diseases share similar features, including genetic susceptibility, the presence of aberrant protein structures, and mitochondrial dysfunction and oxidative stress^6^. Chronic microglia activation, in particular, is a common feature of both AD or PD^7^, and increased levels of inflammatory cytokines have been showed to characterize these conditions^8^, indicating neuroinflammation as an important component in neurodegeneration.

A critical mediator of neuroinflammation is the inflammasome, a multimeric protein complex that induces IL-1beta and IL-18 expression and engages innate immune defenses. The inflammasome can be activated by pathogenic microbes and by endogenous danger signals including protein aggregates, and increasing evidence suggests a roles for excessive inflammasome activation in different neurodegenerative diseases^9^. In particular, one receptor of this system (NOD-like receptor family Pyrin Domain Containing 3, NLRP3), can be activated by Aβ^10^ or by α-syn^11^, creating an inflammatory environment in the brain that contributes to neuronal loss^12^. Notably, NLRP3 up-regulation is now recognized as a central component in the development of several inflammatory and autoimmune diseases^13^.

The molecular mechanisms underlying NLRP3 expression and activation are incompletely understood and it is well known that miRNAs are important post-transcriptional regulators. One of the known negative regulators of NLRP3 expression is miR-223-3p, as indicated by the inverse correlation of expression of this miRNA and NLRP3 activation observed in mononuclear cells^14^. We have recently shown that the NLRP3 inflammasome is activated in Aβ stimulated monocytes of individuals with a diagnosis of AD and mild cognitive impairment (MCI)^15^, a prodromal phase of Alzheimer’s disease. The aims of the study were to verify whether serum miR-223-3p concentration could be used as a biomarker that could facilitate the differential diagnosis of different neurodegenerative diseases (AD, MCI, PD) and to analyze the presence of a possible relationship between miR-223-3p and casapse-1, as an indicator of inflammasome activation.

## Results

### Serum miR-223-3p is down-regulated in AD and MCI individuals but is up-regulated in PD patients

**Figure 1:**
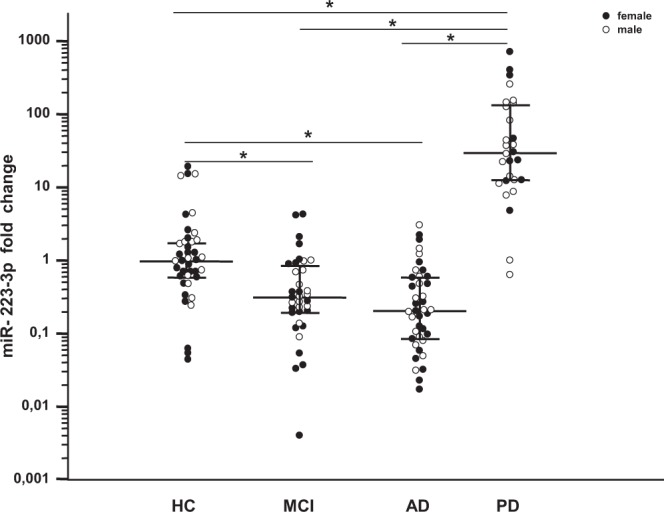
Serum miR-223-3p concentration: miR-223-3p concentration in sera of patients with a diagnosis of mild cognitive impairment (MCI, n = 35), Alzheimer’s Disease (AD, n = 40), or Parkinson’s Disease (PD, n = 28) and in healthy controls (HC, n = 40). Row data were normalized with reference miRNA (*C.el*.miR-39) and ΔΔCt method was used to calculate the fold change relative to HC. Bars represent median fold change and interquartile range (25^th^−75^th^ percentile); statistical significances are indicated. *p < 0.001.

The expression levels of miR-223-3p were evaluated in sera of a group of 143 individuals (40 AD, 35 MCI, 28 PD, 40 HC) using specific qPCR. A significant down-regulation of miR-223-3p serum concentration was observed in the overall group of cognitively impaired patients (AD + MCI) (median fold: 0.26; IQR: 0.12-0.60) compared to HC (0.97; 0.59–1.72 (p < 0.0001). This was confirmed when cognitively impaired patients were stratified according to diagnosis. Thus, a significantly decreased expression of miR-223–3p was observed both in MCI (0.31; 0.19–0.85) and AD (0.21; 0.09–0.59) compared to HC (p < 0.001 in both cases). Notably, and contrary to what was observed in AD and MCI patients, miR-223-3p serum concentration was significantly increased in PD (29.30; 12.43–133.25) compared to HC (p = 0.0006), as well as compared to MCI and AD (p < 0.0001 for both). All these results are showed in Fig. 1. As the Ɛ4 allele of the apolipoprotein E genotype (APOE Ɛ4) is a risk factor for AD, characterization of the APOE Ɛ4 status was performed for all the subjects. As expected, the percentage of APOE Ɛ4 carriers was significantly increased in AD compared to HC but no significant association was observed with miR-223-3p concentration. Finally, no relation was found between miR-223-3p and gender (not shown). All demographic, clinical data and laboratory findings are showed in Table 1.

**Table 1:** Demographic, clinical data and laboratory characterization of the individuals enrolled for this study.

	HC	MCI	AD	PD	Group comparison
***Clinical data***					
Number	40	35	40	28	
Age, years [median; IQR]	75.00 [72.75–78.00]	75.00 [71.00–79.00]	79.00 [73.25–80.75]	74.00 [67.25–80.00]	ns^b^
Gender (M:F)	16:24	14:21	18:22	18:10	ns^c^
MMSE score [median; IQR]	nd	25.7 [24.00–27.08]	20.0 [16.88–22.60]	24.7* [22.58–25.86]	p < 0.0001^b^
Y&H score [median; IQR]	nd	nd	nd	2.5 [2.0–3.0]	
***Laboratory findings***					
*APOE* Ɛ4 Ɛ4-/Ɛ4- (%)	80.0	62.9	47.5	75.0	p = 0.01^c^
Ɛ4-/Ɛ4+	15.0	34.3	45.0	21.4	p = 0.01^c^
Ɛ4 + /Ɛ4+	5.0	2.9	7.5	3.6	ns
*APOE* Ɛ4 carriers^a^, %	20.0	37.1	52.5	24.00	p = 0.0053^c^
miR-223–3p, fold expression [median; IQR]	0.97 [0.59–1.72]	0.31 [0.19–0.85]	0.21 [0.09–0.59]	29.30 [12.43–133.25]	p < 0.0001^b^
Caspase-1, pg/ml [mean ± SEM]	77.20 [9.79]	137.76 [16.32]	102.37 [9.24]	263.52 [20.17]	p < 0.0001^b^

AD, Alzheimer’s disease; MCI, Mild Cognitive Impairment; HC, healthy control; MMSE, Mini Mental State Evaluation; APOE: Apolipoprotein E;

Demographic, clinical and laboratory data are expressed as median and interquartile ranges (IQR) or mean ± standard error of the mean (SEM) as appropriate; ^a^presence of one or two *APOƐ4* alleles; ^b^Kruskal- Wallis test; ^c^Chi-square test, AD *vs* HC. *assessed in a subgroup of 17 PD patients.

### Relation between serum miR-223-3p levels and cognitive decline (MMSE)

Severity of cognitive decline was determined by evaluating the MMSE score of all AD, MCI included in the study. Based on these evaluations, patients were defined as being affected by a moderate (MMSE > 15; n = 34) or severe (MMSE ≤ 15; n = 6) cognitive decline; in MCI individuals cognitive decline was, as per definition, mild.

**Figure 2:**
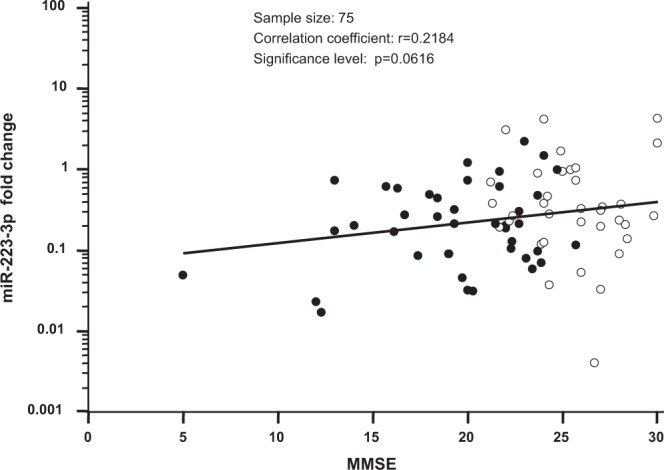
Correlation between miR-223-3p concentration and MMSE: scatter plot showing relationship between MMSE score and miR-223-3p concentration in serum obtained from Alzheimer’s disease (AD, represented by black dots) and mild cognitive impairment (MCI, represented by white dots) subjects.

Results showed that compared to HC, miR-223-3p serum concentrations decreased progressively, being higher in MCI individuals (median MMSE: 25.7) (miR-223-3p: 0.31; 0.19-0.85; p = 0.0002); intermediate in moderate AD patients (median MMSE: 21.7) (miR-223-3p: 0.21; 0.09–0.60; p < 0.0001) and lower in severe AD patients (median MMSE: 12.60) (miR-223-3p: 0.11; 0.02–0.20; p = 0.0016). Analysis of the direct correlation between MMSE score and miR-223-3p serum concentration approached but did not reach significance (p = 0.062; r^2^ = 0.218), probably due to limited number of subjects with MMSE lower than 15 (n = 6) (Fig. 2).

Regarding PD patients, based on clinical observation of cognitive deficit, MMSE scores were evaluated in 17 patients; only 3 PD patients were severely compromised (<15), whereas the others (PD-MCI) showed mild cognitive decline (median MMSE: 25.05), comparable to value observed in MCI subjects. To note, the miR-223-3p serum concentration in PD-MCI (median fold: 36.73; IQR 12.68–143.31) remains up-regulated compared to HC (p < 0.0001) and was significantly higher compared to MCI and AD (p < 0.0001 for both).

### Serum concentration of caspase-1

**Figure 3:**
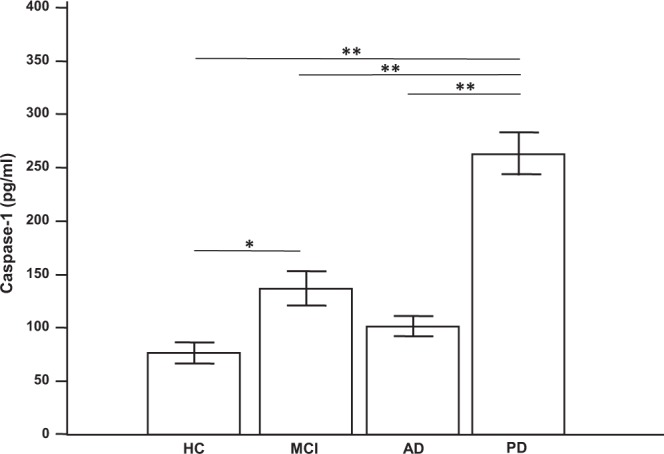
Serum caspase-1 concentration: caspase-1 concentration in sera of patients with a diagnosis of mild cognitive impairment (MCI, n = 35), Alzheimer’s Disease (AD, n = 40), or Parkinson’s Disease (PD, n = 28) and in healthy controls (HC, n = 40). Means, standard error of the mean (SEM) and statistical significances are indicated. *p = 0.0033; **p < 0.0001.

NLRP3 inflammasome activation was indirectly inferred by measuring serum concentration of caspase-1 (aka IL-1β-converting enzyme), a protease that, following the functional assembly of the NLRP3 inflammasome, cleaves the IL-1β cellular inactive precursor to extracellular active form. Results showed that, as compared to HC, caspase-1 serum concentration was significantly increased in MCI (p = 0.0033) and PD (p < 0.0001) and, although not significantly, also in AD patients (p = 0.068) (Fig. 3).

### Diagnostic value of miR-223-3p and caspase-1 in serum (ROC analysis)

**Figure 4:**
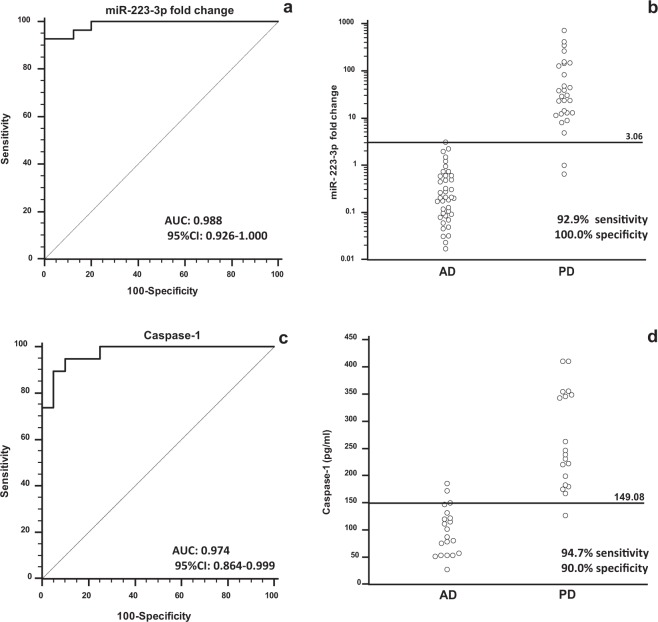
ROC analysis: Receiver Operating Characteristic (ROC) curve analysis (panel **a,c**) and dot diagram (panel **b,d**) showing discriminative power of miR-223-3p fold change (panel **a,b**) or caspase-1 (panel **c,d**) in serum between Alzheimer’s Disease (AD) and Parkinson disease (PD) patients.

Receiver Operating Characteristic (ROC) curve analysis was used to evaluate the diagnostic accuracy of serum miR-223-3p and caspase-1 as possible non-invasive biomarkers in neurodegenerative conditions. The area under curve (AUC) analysis showed that miR-223-3p serum concentration clearly discriminate between: 1) MCI and HC (AUC = 0.747; 95% CI = 0.634–0.840); 2) AD and HC (0.844; 0.771–0.900)*;* 3) PD and HC (0.934; 0.846–0.980), and 4) AD and PD (0.988; 0.926–1.000) (Fig. 4, panel a and b).

The same analyses were performed to verify possible predictive values of caspase-1 serum concentration. Results showed a good predictive value allowing this parameter to discriminate between MCI and HC (0.800; 0,644 to 0.909), and AD and PD (0.974; 0.864 to 0.999) (Fig. 4, panel c and d). These findings suggest that miR-223-3p could be possible useful biomarkers for the diagnosis of these neurodegenerative diseases and to allow the early discrimination between AD and PD patients.

## Discussion

To evaluate possible changes of miR-223-3p serum concentration in patients with diverse neurodegenerative conditions, qPCR assay was performed in a cohort of patients with a diagnosis of either MCI, AD or PD as well as in cognitively normal controls. Results showed that serum concentration of miR-223-3p is significantly reduced in individuals with cognitive decline (AD and MCI) compared to normal controls; these data confirm previous results obtained in AD individuals, showing a significant downregulation^16,17^ of miR-223-3p in serum.

Interestingly, miR-223-3p expression decreased in accordance to the severity of disease as measured by MMSE: these results, in accordance with other previous findings^16,17^, suggest that miR-223-3p might be a useful and easily measurable biomarker for cognitive decline. Our study indicates that in MCI, a condition that is often the prodromal phase of Alzheimer’s disease, miR-223-3p serum concentration is already significantly reduced compared to control subjects.

The results obtained in PD groups are surprisingly: in contrast to what we observed in AD and MCI, miR-223-3p serum concentration was significantly increased in PD patients; moreover it is important to underline that a significant up-regulation of miR-223-3p was observed even considering alone those PD patients showing cognitive decline (PD-MCI) compared to MCI subjects, suggesting a different pathogenic mechanism underlying cognitive decline in PD and MCI/AD.

The cognitive decline is the most prevalent non-motor symptoms in PD patients and it may cause misdiagnosis, especially in the early stage of diseases. The underlying mechanism of cognitive impairment is not yet fully elucidated and different factors (neurodegeneration, neuroinflammation, neurotrophic and genetic factors) can concur to determine this clinical phenotype. A recent meta-analysis^18^ highlights the need of combined biomarkers that can better reflect the complexity of pathological processes correlated to cognitive dysfunction in PD; our study suggests that, besides to molecular imaging that measures neurodegeneration, protein accumulation (PET), abnormalities of brain function and connectivity, also microRNA should be included in this approach to facilitate the early diagnosis of PD subjects with mild cognitive decline; future studies evaluating changes of miR-223-3p over time will be important to understand better the utility of this biomarker for monitoring cognitive deterioration and also for evaluating cognitive rehabilitation effect.

In our study the discriminative power of circulatory miR-223-3p is also showed by ROC curve analysis, suggesting that measure of its concentration might be considered as non-invasive biomarker useful for differential diagnosis between AD/MCI and PD.

In the attempt to better understand the biological meaning of these results, we then analyzed the possible relationship between miR-223-3p and the NLRP3 inflammasome activation. The background for these analyses stems from a number of recent reports indicating inflammation as a pivotal component in neurodegeneration; in particular, accumulating evidences support the involvement of NLRP3 inflammasome in the initiation or progression of neurodegenerative conditions including AD and PD^15,19^. The link between NLRP3 and miR-223-3p was given by the observation that this molecule was shown to play an important role as a post-transcriptional regulator of NLRP3^14^. NLRP3 inflammasome activation was analyzed by measuring serum concentration of caspase-1, a protein required for the maturation and secretion of the pro-inflammatory cytokines interleukin (IL)-18 and IL-1β^9^.

Results showed that caspase-1 concentration was significantly increased in serum of MCI and PD patients and, although not significantly, in AD, indicating that the NLRP3 inflammasome is indeed activated in these patients affected by neurodegenerative conditions.

The presence of increased caspase-1 and miR-223-3p may appear in contrast with the negative regulatory role of miR-223 on NLRP3 expression in monocytes^14^: although it is difficult to interpret these findings, it is to note that the nature of the relationship between extracellular (serum) and intracellular miR-223 concentration is not known; moreover, miRNA mediated target regulation is cell-type dependent and miR-223 concentration in serum derives only partially from monocytes, as miRNAs can originate from different cell types (peripheral or brain cells), can derive from the drainage of CSF to the periphery, can be the result of different mechanisms, including vesicles secretion and release by dying cells, and, finally, can be also selectively released in particular pathophysiological states as showed, for example, in monocytes after different stimuli^20^; other inflammasome receptors, at now not well characterized, can contribute to caspase-1 production, i.e. NLRP1 or NLRC4^9^: it is possible that more than one inflammasome-forming protein, in response to a multitude of signals which occur during neuroinflammation (protein aggregates, oxidative stress, mitochondrial DNA release etc.), can add complexity to this scenario for the different neurodegenerative diseases.

Our results seem to indicate that miR-223-3p levels measured in PD serum could be related to mechanisms different from inflammasome. It is known that miR-223-3p, modulating many other targets involved in differentiation/proliferation (i.e.NFI-A; /EBPbeta, Mef2c) and NF-kB pathways (i.e. STAT3, IKK), may have cell and tissue intrinsic effects in different pathologies, as inflammatory disorders, infection diseases and cancers^21^. Moreover, targeting glutamate receptors and modulating calcium influx and neuronal excitability, miR-223-3p can have a neuroprotective effect in neuronal cells^22^. As the miR-223 is involved in a broad range of cellular processes supposed to play a role on the pathogenesis of dementia, it is also plausible that the different miR-223-3p concentration in serum we observed can be due to all these components.

Moreover many other elements inside the cells can regulate the activation of the NLRP3 inflammasome, i.e. miR-7, another negative regulator of NLRP3^23^, that was shown to be significantly reduced in PD brain^24^. The decreased expression of miR-7 is able to increase the expression of alpha-synuclein^25^, and at the same time it may lead in a defective control over NLRP3 inflammasome activation in PD; it is tempting to speculate that the augmented amounts of miR-223-3p we observed in these patients could be an attempt to balance such excessive chronic activation.

It is also interesting to note that a previous work^26^ showed that the expression of miR-223-5p, the strand which is opposite and complementary to the miR-223-3p we analyzed, is up-regulated in serum of PD individuals. These findings, together with our results, suggest that the biosynthesis of precursor-miR-223 (pre-miR-223) is increased in PD patients, an intriguing hypothesis that has to be verified.

Although at now it is impossible to draw conclusion on mechanistic aspects, our work clearly indicates that miR-223-3p deserves further exploration about the reason for deregulation in serum of neurodegenerative diseases, the functional effect and the relation with caspase-1concentration.

Whether transcriptional factors known to be involved in the biogenesis of miR-223 (PU.1, C/EBP, NFI-A) can determine the different expression of miR-223 in serum of AD or PD patients still remains an open question to investigate.

The identification of critical steps in molecular mechanism underlying the disease-associated neuroinflammation will create the conditions for novel therapeutic approaches to modulate inflammasome activity in neurodegenerative diseases. In the meanwhile, the future efforts should be pointed on validation of reliable biomarkers, including miRNAs, for a more accurate identification and monitoring of cognitive impairment.

On the whole, although these results will need to be validated with large-scale clinical cases, our findings indicate that serum concentration of miR-223-3p discriminates between AD, MCI and PD patients and healthy controls, and suggest that this molecule could be used as a non-invasive biomarkers for the differential diagnosis of these conditions and to evaluate the progression of dementia.

## Methods

### Patients and controls

One-hundred-forty-three individuals (40 AD, 35 MCI, 28 PD and 40 HC) were recruited for this study from the Rehabilitative Neurology Unit at the Don Carlo Gnocchi Foundation in Milano (Italy). The diagnosis of probable AD was performed according to the NINCDS-ADRDA criteria^27^ and to the updated guidelines for AD of the National Institute on Aging Alzheimer’s Association^28^.

MCI individuals had to fulfill the Petersen^29^ and Grundman^30^ operational criteria. Diagnosis of PD was based on the Queen Square Brain Bank Criteria^31^. Disease stage has been defined for all the PD according to modified Hoehn and Yahr (H&Y) criteria^32^. All but one PD subjects received dopaminergic treatment at the moment of sampling; HC were selected according to the SENIEUR protocol for immuno-gerontological studies of European Community’s Control Action Program on Aging^33^. Cognitive status was assessed by Mini-mental state examination (MMSE, score for inclusion as normal control subjects ≥28). The present study conforms to the principles of Helsinki Declaration. All experimental protocols were approved by Don Gnocchi Foundation and all methods were carried out in accordance with the guidelines of the ethic committee of the Don Gnocchi Foundation. All patients or their legal guardians gave informed consent, according to a protocol approved by the local ethics committee of the Don Carlo Gnocchi Foundation in Milano, Italy.

Demographic and clinical characteristics of the enrolled subjects are reported in Table 1.

### Samples collection

Peripheral blood was collected from all subjects enrolled in the study. Serum was separated within 1 h from blood draw by centrifugation at 1500 *g* for 10 min at room temperature. The clear supernatant was aliquoted into RNase/DNase-free tubes and stored at −80 °C until use. Genomic DNA was isolated from whole blood from each subject by phenol-chloroform extraction.

### *APOE* genotyping

Customer-design Taqman probes for the 112 and 158 codons were used to determine the genotype of apolipoprotein E gene (*APOE*)^34^.

### Serum miRNA isolation and reverse transcription

Total RNA was extracted from 200 µl of serum using spin column chromatography (miRNeasy Mini kit, Qiagen GmbH, Hilden, Germany) as previously reported^35^. One μg of MS2 bacteriophage RNA (Roche Life Science, Mannheim, Germany) and 5 μl of 5 nM synthetic spike-in, *Caenorhabditis elegans*-miR-39 (*C.el*. miR-39-3p) were added to the samples during processing, according to the manufacturer’s protocol. The inclusion of the synthetic miRNA was necessary for normalization of sample-to-sample variation in RNA isolation. RNA was eluted by adding 15 µL of RNase-free water, and 4 µl was subjected to retro-transcription using Universal cDNA synthesis kit II (Exiqon Inc., Vedbaek, Denmark) in a total of 20 µl, including 1 µl of synthetic spike-in UniSp6. To avoid variation due to sample differences and handling, all variables were kept consistent throughout the study. The amount of RNA in serum is very low, and the concentration could not be accurately determined in these samples. Consequently, for the qPCR analysis, a constant input amount was used for all samples, using the same starting volume rather than RNA quantity. The uniformity of the RNA extraction and efficiency of the RT and PCR reactions were analyzed monitoring the synthetic spike-in RNA templates.

### Measurement of miR-223-3p levels by qPCR

Sera of 143 subjects were collected for analysis by specific qPCR. cDNA (20x diluted) was assayed in 10 µl PCR reaction containing specific LNA™-individual microRNAs assay (Exiqon) for detection by single qPCR of target miRNAs (miR-223-3p, cat. 205986) and of synthetic spike-in (C.el. miR-39-3p, Exiqon, cat. 203952) as reference miRNA. We have previously used this approach in the field of circulating miRNAs^35^.

Absence of qPCR inhibition for haemolysis was verified monitoring the stability of Cq of miR-16-5p (Exiqon, cat. 204409) commonly found in red blood cells. cDNA was tested in triplicate in qPCR; negative controls without rt-template and no template controls were included in each sessions. qPCR amplification was performed with a real-time PCR system (StepOne, Life Technologies, Carlsbad, CA, US) as previously reported^35^; melting curve was used to evaluate the specificity of the amplification products. Baseline and threshold were set manually, and Cq analysis was accomplished with StepOne^TM^ software (Ver. 2.1).

### Cytokine assay

Caspase-1(p20) serum concentration was determined by enzyme linked immunoassay (ELISA), using commercially available kit (Quantikine ELISA, Duoset ELISA, R&D Systems, Minneapolis, MN, US). The kit sensitivity was 1.24 pg/ml.

### qPCR data and statistics

qPCR row data were normalized using exogenous spike-in C.el.miR-39-3p as reference. Relative quantification was determined by the comparative delta-Cq method (ΔΔCq) using qBase + software (version 3.0, Biogazelle, Belgium)^36^. Results are expressed as fold expression changes relative to HC.

Statistical analyses were accomplished using commercial software (MedCalc®, version 11.5.0.0). Categorical data were compared using Chi-square test. Continuous variable, are reported as mean and standard error of the mean (SEM) or as median and interquartile ranges (IQR: 25th and 75th percentile). Parametric (ANOVA and T-test) or non-parametric tests, (Kruskal–Wallis, Mann-Whitney test) were used to determine the significance between groups. miRNA relative expression were analyzed after logarithmic transformation. Spearman’s rank correlation coefficient was used in the correlation analysis between laboratory data. P values < 0.05 were considered statistically significant. Receiver operating characteristics analysis (ROC) and area under curve (AUC) were used to evaluate the potential of miRNA as biomarker.

## Data Availability

The datasets supporting the conclusions of this article are included within the article.
